# Oceanic states of consciousness—an existential-neuroscience perspective

**DOI:** 10.3389/fnhum.2025.1653801

**Published:** 2025-08-11

**Authors:** Human-Friedrich Unterrainer

**Affiliations:** ^1^Faculty of Psychotherapy Science, Sigmund Freud Private University, Vienna, Austria; ^2^Institute for Religious Studies, University of Vienna, Vienna, Austria; ^3^ARH - Addiction Research Hub, Grüner Kreis Ltd., Vienna, Austria; ^4^University Department of Psychiatry and Psychotherapeutic Medicine, Medical University of Graz, Graz, Austria

**Keywords:** affective neuroscience, ego dissolution, existential neuroscience, oceanic feeling, mysticism, psychoanalysis, psychedelic therapy

## Abstract

Oceanic states of consciousness—characterized by ego dissolution, unity, and timelessness—have long occupied a liminal space between psychopathology and transcendence. This paper explores these states through the interdisciplinary lens of existential neuroscience, integrating insights from psychoanalysis, existentialism, affective neuroscience, and psychedelic research. Starting with the psychoanalytic tension between Freud’s view of the oceanic feeling as a regressive illusion and Jung’s framing of it as a transformative encounter with the unconscious, this paper examines how creative and mystical experiences often arise from this dissolution of self-boundaries. Drawing on art theorist Anton Ehrenzweig and examples from figures like Vincent van Gogh and Antonin Artaud, I highlight how oceanic states may catalyze both visionary insight and psychological disintegration. Neuroscientific models, including the REBUS theory and studies of the Default Mode Network (DMN), suggest that ego dissolution reflects a flexible reorganization of brain function rather than dysfunction. The Peri-Aqueductal Gray (PAG), a midbrain structure associated with affect regulation and spiritual experience, emerges as a key neural hub linking primal affective states with mystical awareness. Existential thinkers such as Sartre, Heidegger, and Merleau-Ponty provide a philosophical framework for interpreting these phenomena as moments of existential rupture and potential authenticity. Oceanic states thus challenge conventional notions of the self as fixed and bounded. Rather than categorizing them as pathological or purely mystical, it is proposed here that these states represent affectively charged boundary experiences - ones that require contextual integration and offer deep insight into the nature of selfhood, meaning, and transformation.

## Introduction

The desire to transcend the bounded ego and dissolve into a larger, timeless unity has echoed through human cultures and philosophies for millennia. In ancient Greek tradition, ekstasis—literally “standing outside oneself”—described a form of divine madness linked to Dionysian ritual and the mystical insight of early thinkers like Heraclitus. In modern thought, this longing to transcend the boundaries of the self was taken up by Romain Rolland, who described the “oceanic feeling” as a sensation of eternity and unity with the world as a whole (Parsons, 1999). Rolland communicated this idea to Freud, who discussed it in Civilization and Its Discontents—only to dismiss it as a regressive illusion rooted in early infantile consciousness (Freud, 1930; Harrison, 1979). In contrast, Jung (1969) interpreted such states not as pathological regressions but as transformative encounters with the collective unconscious -often essential to the individuation process. Similarly, art theorist Ehrenzweig (1967) viewed the temporary breakdown of structured ego boundaries as central to artistic creation and deeper psychic integration. These contrasting views reflect a long-standing tension: are oceanic states of consciousness regressive breakdowns of ego structure, or affectively charged openings to deeper dimensions of human experience?

This paper explores oceanic states through the interdisciplinary lens of existential neuroscience, integrating psychoanalytic, philosophical, and neuroscientific insights. Special attention is given to how such states—arising through meditation, psychedelics, or spontaneous mystical episodes—may reflect a dynamic reorganization of self-processing rather than psychopathologys. This synthesis aims to contextualize oceanic experiences as transitional phenomena that challenge fixed models of the self and offer transformative potentials for insight, healing, or collapse.

### Oceanic feelings and the collective unconscious

While Freud interpreted oceanic feelings as regressive, Jung’s analytical psychology provides a more integrative perspective. Although Jung did not explicitly use the term “oceanic feeling,” his accounts of mystical experiences, encounters with the Self, and ego dissolution during the process of individuation resonate strongly with Rolland’s description. For Jung (1969), such states emerge when the ego engages with the deeper layers of the collective unconscious, often triggered by symbolic or numinous material. He viewed the numinosum a concept adopted from Otto’s (1923) notion of the *mysterium tremendum et fascinans* as central to understanding spiritual experience: an encounter marked by awe, transcendence, dread, and fascination. Far from being pathological, these moments signal the psyche’s attempt to achieve wholeness, bridging the ego and the archetypal matrix of the unconscious. In contrast to Freud, oceanic states thus become markers of transformation rather than regression—essential components of the individuation process and of personal development (Jung, 1969).

### Oceanic feeling, creativity, and the threshold of the unconscious

Expanding beyond classical psychoanalytic interpretations, Anton Ehrenzweig—an Austrian-born art theorist and psychoanalyst—offered a compelling view of oceanic feelings as central to the creative process. In The Hidden Order of Art, Ehrenzweig (1967) argued that artistic insight often arises through a temporary collapse of structured, linear thought. This disintegration, far from pathological, allows for a more fluid and undifferentiated mode of perception, in which the unconscious mind can reorganize fragmented impressions into meaningful wholes. He associated this state with the oceanic feeling—a moment of perceptual and psychic boundlessness, in which ego boundaries dissolve to reveal a deeper psychic order. For Ehrenzweig, such moments are not regressive but generative, enabling access to layers of meaning inaccessible to rational cognition (Saarinen, 2014). This interpretation opens a broader view of the oceanic feeling as a liminal state—one that may give rise to both intense creativity in line with profound disintegration.

The artistic lives of figures like Louis Wain, Vincent van Gogh, and Antonin Artaud illustrate this tension. Wain’s progressively abstract cat portraits, with their swirling patterns and collapsing form, suggest a loss of structured reality—a perceptual fusion evocative of psychotic fragmentation and oceanic immersion (Robins, 1992). Vincent van Gogh, in works such as The Starry Night, channeled intense sensory and emotional energy into visionary landscapes that hover between ecstatic transcendence and psychic instability (Blumer, 2002). Antonin Artaud, through his theatrical and literary explorations, described states of cosmic absorption that closely resemble oceanic experiences—though often accompanied by terror, dread, and disintegration (Artaud, 1995). These cases highlight the precarious and paradoxical nature of oceanic states: not merely mystical or redemptive, but psychologically charged experiences situated at the unstable boundary between visionary insight and psychic collapse (Fink et al., 2014).

### Existentialist philosophy and the boundaries of the self

Existentialist thinkers have long grappled with the nature of consciousness and the self, providing a powerful framework for interpreting oceanic states. Sartre (1956) famously argued that the self is not a static entity but a project—an ongoing act of self-transcendence. For Sartre, to exist is to commit oneself in a continual movement toward the future, in the shadow of freedom and responsibility. The experience of ego dissolution, then, could be seen not as a loss of identity, but as a confrontation with the nothingness that grounds consciousness itself. Martin Heidegger’s existential ontology deepens this perspective.

In Being and Time, Heidegger (1962) presents Dasein—the human being—as fundamentally being-toward-death, situated in a world of meanings, always already embedded in Being. Moments of boundary dissolution, as found in oceanic states, may act as existential ruptures -interruptions of everyday existence—that allow for authentic disclosure of Being. Rather than escapism, these experiences might constitute radical awakenings to the groundlessness and mystery of existence itself. Merleau-Ponty (2002), finally, reframed the self not as a disembodied ego, but as an embodied subject embedded in the world. His notion of pre-reflective consciousness suggests that the self is not originally isolated from the world but co-emerges with it in perception. Oceanic feelings, especially those induced through meditative or psychedelic practice, might reflect a return to this original unity—a loosening of dualistic boundaries that reveals a more primordial, embodied mode of being. Together, existentialist perspectives invite a reconsideration of oceanic states not as aberrations, but as meaningful experiences that reveal the dynamic and constructed nature of the self (see also Barrett, 1958 for an enhanced introduction).

While existential and phenomenological thinkers in the West—such as Heidegger, Sartre, and Merleau-Ponty emphasized the fluid, embodied, or projective nature of the self, similar insights have long existed in non-Western traditions. Buddhist philosophy, in particular, posits that the self is not a stable entity but a transient construct—an idea central to the doctrine of anattā (non-self). Contemporary philosophers like Metzinger (2003) have explicitly acknowledged this lineage, arguing that the notion of the self as a model or process rather than a substance has roots in Buddhist contemplative frameworks.

### Oceanic feelings and the neuroscience of ego dissolution

Recent advances in cognitive neuroscience have revitalized interest in altered states of consciousness, particularly those involving ego dissolution, self-transcendence, and mystical unity. Neuroimaging studies of meditation, psychedelic experiences, and flow states have identified recurring patterns of brain activity that correlate with the phenomenology of oceanic feelings (Brewer et al., 2011; Carhart-Harris et al., 2014). The default mode network (DMN) is a large-scale brain network primarily composed of the dorsal medial prefrontal cortex, posterior cingulate cortex, precuneus, and angular gyrus. Central among recent findings is the DMN’s role in self-referential processing, autobiographical memory, and mind-wandering, highlighting its involvement in internally directed cognitive functions (Raichle et al., 2001). Under the influence of substances like psilocybin, lysergic acid diethylamide (LSD), and N, N-dimethyltryptamine (DMT), activity in the DMN becomes significantly reduced or decoupled, corresponding to subjective reports of ego dissolution, boundary loss, and feelings of unity with the cosmos (Carhart-Harris et al., 2013, 2014), a finding supported by numerous neuroimaging studies (Lebedev et al., 2015; Nour and Carhart-Harris, 2017; Palhano-Fontes et al., 2015; Pasquini et al., 2020; Tagliazucchi et al., 2016). To explain these results, Carhart-Harris and Friston proposed the REBUS model (Relaxed Beliefs Under Psychedelics), suggesting that psychedelics work by flattening hierarchical priors in the brain’s predictive coding system. This temporary breakdown of top-down constraints permits a state of heightened neural entropy and cognitive flexibility—conditions under which previously suppressed emotional or cognitive content may surface and be restructured (Carhart-Harris and Friston, 2019; see also Albert and Back, 2025). Building on this, the Altered Beliefs Under Psychedelics (ALBUS) model suggests that psychedelics can lead to both belief relaxation and reinforcement, depending on factors such as dosage, set and setting, and their modulation of 5-HT2A receptor signaling (Safron et al., 2025). While the ALBUS model offers one promising framework, other explanatory approaches highlight the conceptual diversity in current psychedelic science—such as Gallimore’s (2015) integrated information theory, or the view proposed by James et al. (2022), who question whether psychedelic (oceanic) experiences are the result of altered information processing in the brain or of reduced filtering mechanisms that temporarily reveal a fundamental conscious reality normally obscured by everyday neurophysiological constraints. These neuroscientific frameworks are complemented by psychometric models that seek to quantify altered states of consciousness. One of the most widely used is the Altered States of Consciousness Rating Scale (ASC; Studerus et al., 2010), which includes “Oceanic Boundlessness” as a core dimension. This factor captures feelings of unity, dissolution of ego boundaries, blissful states, and altered perception of time and space—phenomena that align closely with both mystical experiences and the subjective effects of psychedelics. The ASC framework also includes opposing dimensions, such as Anxious Ego Dissolution, helping differentiate between positive and destabilizing forms of ego-loss.

**Figure 1:**
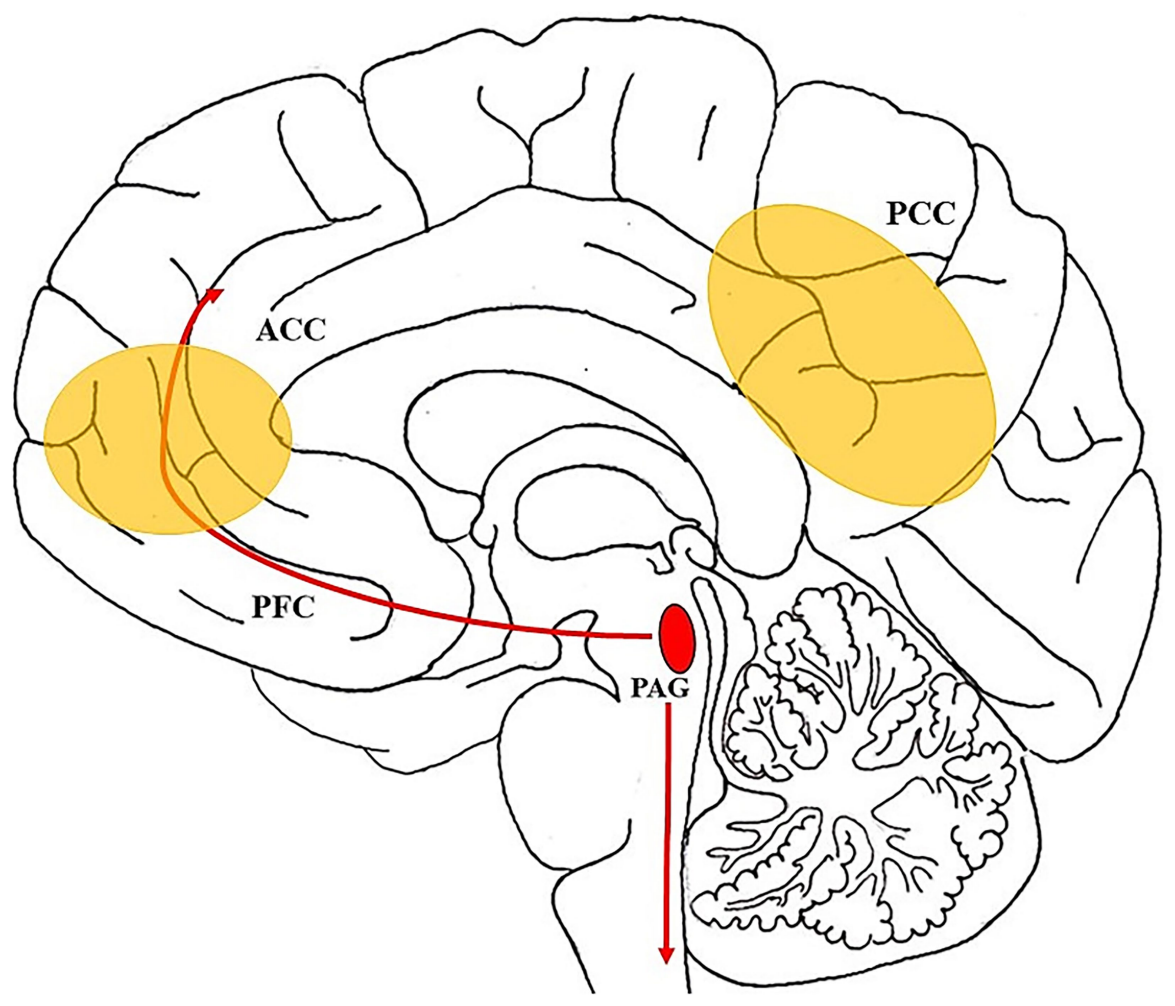
*“The Oceanic Wave of Consciousness”*. This schematic illustrates the central role of the periaqueductal gray (PAG) in mediating oceanic states of consciousness. Shown in red, the PAG is a midbrain hub involved in affect regulation, pain modulation, and autonomic control. Arrows represent ascending projections to the anterior cingulate cortex (ACC) and prefrontal cortex (PFC), as well as descending pathways to the brainstem and peripheral systems. These connections reflect the PAG’s integrative function in generating core affective states (Alcaro et al., 2017; Solms and Panksepp, 2012), which may underpin experiences of ego dissolution and emotional unity. Recent evidence links the PAG to spiritual and existential experiences (Ferguson et al., 2022), suggesting that such states arise from embodied affective dynamics. As Saarinen (2014) argues, these “existential feelings” are phenomenologically distinct from Panksepp’s (2004) primary emotions, pointing to a layered structure of affective consciousness. Oceanic states may thus represent a wave-like dynamic—rising from subcortical affective cores, integrating through cortical systems, and resonating through bodily perception. In yellow color: Main brain areas of the default mode network (DMN): Medial Prefrontal Cortex (mPFC) and Posterior Cingulate Cortex (PCC); simplified illustration.

Furthermore, research from affective neuroscience, particularly the work of Jaak Panksepp, supports the idea that oceanic feelings engage primal emotional circuits in the subcortical brain (Alcaro et al., 2017; Hiebler-Ragger et al., 2018). Panksepp’s seven primary emotional systems—SEEKING, CARE, PLAY, LUST, FEAR, RAGE, and SADNESS—represent evolutionarily conserved affective networks that underlie mammalian emotional life (Panksepp, 2004). While oceanic feelings are often linked with spirituality, they need not be religious; they also appear in creative insight, aesthetic absorption, or non-theistic awe (Yaden et al., 2017). Recent findings suggest that such experiences may be accompanied by aesthetic chills, which reliably co-occur with feelings of self-transcendence and unity (Christov-Moore et al., 2024). These states do not map neatly onto any single emotional system but may arise from the integration or modulation of circuits like SEEKING and CARE during altered states of consciousness (see, e.g., Solms, 2021 for an in-depth discussion). The affective core of the self, as theorized by Alcaro et al. (2017) (see also Figure 1), provides a neuro-archetypal foundation for such experiences, rooted in deep midline structures such as the periaqueductal gray (PAG)—a region associated with emotional regulation, spiritual feelings, and existential awareness (Ferguson et al., 2022). Importantly, oceanic feelings have been discussed separately from Panksepp’s discrete primary emotions, as they may reflect broader existential affective tones rather than specific motivational systems (Saarinen, 2014). These distinctions have informed the development of psychometric tools like the OCEANic Scale, which captures individual tendencies toward both the blissful and terrifying aspects of ego-dissolution (Schmautz et al., 2024; Straßnig et al., 2025). The emerging consensus in neuroscience is that oceanic experiences are not inherently pathological, but instead reflect a dynamic reconfiguration of self-processing—which can be therapeutic, destabilizing, or both, depending on context, integration, and meaning-making (Letheby and Gerrans, 2017).

### The periaqueductal gray: neural nexus of oceanic experience

Located in the midbrain, the periaqueductal gray (PAG) has traditionally been studied in the context of pain modulation, autonomic control, and survival responses (see also Figure 1 for further illustration). However, recent findings have implicated the PAG in a much broader array of functions, including emotional regulation, attachment, and spiritual or mystical experiences (Ferguson et al., 2022; Linnman et al., 2012; Unterrainer, 2025a). In particular, lesion network mapping studies have identified the PAG as a central node in the neural circuit of spirituality, correlating with traits such as awe, ego-dissolution, and altered self-awareness. These are precisely the affective and perceptual dimensions associated with oceanic states of consciousness. The PAG’s involvement in both threat defense and care-related affect suggests that it operates at a basic, embodied level of experience—where boundaries between self and world, safety and surrender, can be radically reorganized. This is especially salient in mystical or psychedelic states, where feelings of unity, timelessness, and transcendence may coexist with dread, ego death, or annihilation. From this perspective, the oceanic feeling may not emerge from purely cortical or abstract cognitive processes, but from a subcortical, somatic re-tuning of affective processing—rooted in evolutionarily ancient neural regions like the PAG (see Alcaro et al., 2017). Furthermore, in oceanic states, the DMN may loosen its regulatory grip, allowing subcortical affective signals from the PAG to rise into awareness. This bottom-up/top-down interplay may underlie the unique blend of visceral intensity and ego transcendence that characterizes mystical or creative states of consciousness (Carhart-Harris et al., 2014).

This insight carries significant implications for creativity and the psychotic spectrum, long understood to exist in a delicate tension (Claridge, 1997; Unterrainer and Lewis, 2014). The same threshold affective shifts that open access to transpersonal or archetypal experience may also render the ego vulnerable to disintegration. The periaqueductal gray (PAG), by mediating the basic feeling-tone of being alive—through fear, care, awe, or surrender—may play a key role in facilitating this affective threshold crossing (Panksepp, 2004). Indeed, as seen in artists like Vincent van Gogh, Louis Wain, and Antonin Artaud, the collapse of structured perception into swirling, symbolic, or ecstatic forms often mirrors descriptions of both mystical union and psychotic fragmentation (Laing, 1967). These cases exemplify what Koestler (1964) termed the “act of creation” as a “bisociation”—a clash between habit and insight. In this model, oceanic states may represent moments of neural and psychic instability where new configurations of meaning can emerge, but only at the risk of losing contact with consensus reality. Thus, the PAG may be understood as a neurobiological pivot between embodiment and transcendence, between the affective core self (Panksepp, 2004) and the symbolic re-imaginings of self found in art, mysticism, and madness. It offers a potential somatic foundation for what Ehrenzweig saw as the integration of unconscious form, and for what Jung described as the numinous eruption of the Self.

## Discussion

Oceanic states of consciousness—marked by ego dissolution, emotional intensity, and a sense of unity—occupy a unique intersection of philosophy, psychology, neuroscience, and art. They have been variously described as regressive illusions (Freud, 1930), mystical insights (James, 1902), archetypal encounters (Jung, 1969), or creative ruptures (Ehrenzweig, 1967). Increasingly, neuroimaging and affective neuroscience suggest these are not metaphysical anomalies but arise from altered brain dynamics, particularly involving the default mode network (DMN), limbic circuits, and periaqueductal gray (PAG) (Carhart-Harris et al., 2013; Ferguson et al., 2022). From an existential neuroscience perspective, oceanic experiences challenge static models of selfhood. Philosophers like Sartre and Heidegger conceptualized the self as a contingent project, always unfolding and shaped by its relation to finitude. Neuroscience now supports the idea that egoic processing is dynamically constructed and modulated by predictive top-down models (Carhart-Harris and Friston, 2019). Ego dissolution, in this view, is not inherently pathological but reflects a shift in the self’s structure—opening space for trauma, transformation, or transcendence. While current models increasingly interpret oceanic experiences as dynamic reconfigurations of self-processing rather than pathological states, no single explanatory framework has achieved broad consensus. Competing approacheshighlight the theoretical openness of this domain (Gallimore, 2015; James et al., 2022). Continued research is needed to clarify the mechanisms underlying these states. At the same time, the unique phenomenology of oceanic experiences may offer valuable insights into the structure of consciousness itself.

### Clinical potentials and existential risks

This fluidity has both clinical and creative applications. Psychedelic-assisted psychotherapy, meditation, and transpersonal approaches increasingly use oceanic states to break rigid cognitive patterns, process trauma, and restore a sense of connectedness (Ko et al., 2022; Roseman et al., 2018). These interventions mirror psychodynamic insights: unconscious material must surface to be integrated. The OCEANic Scale developed by our group (Schmautz et al., 2024) reflects this duality, capturing both the bliss and terror of ego-transcendence. While mystical unity and psychotic fragmentation may appear phenomenologically similar, they are existentially distinct. However, these states also carry risks. For every instance of healing, there are reports of derealization, spiritual crisis, or psychotic collapse (Grof and Grof, 1989). The PAG—central to survival circuits and affective tone—may act as a neurobiological gatekeeper between psychic coherence and chaos (Damasio and Carvalho, 2013).

## Conclusion

Figures like van Gogh, Wain and Artaud illustrate how oceanic immersion can yield sublime creativity while exposing psychic vulnerability. This underscores the need for integrative frameworks—therapeutic, philosophical, and communal—that can help contain and contextualize these experiences. Oceanic states lie at a threshold: between ego and cosmos, insight and dissolution, healing and breakdown. Viewed through existential neuroscience, they are not aberrations but liminal openings. As I noted in a previous paper (Unterrainer, 2025b), these moments demand not only clinical understanding but existential care: they reveal how the mind, when unmoored, may encounter something beyond itself—and must choose how to respond.

## Data Availability

The original contributions presented in the study are included in the article/supplementary material, further inquiries can be directed to the corresponding author.
